# Fagaceae tree species allocate higher fraction of nitrogen to photosynthetic apparatus than Leguminosae in Jianfengling tropical montane rain forest, China

**DOI:** 10.1371/journal.pone.0192040

**Published:** 2018-02-01

**Authors:** Jingchao Tang, Ruimei Cheng, Zuomin Shi, Gexi Xu, Shirong Liu, Mauro Centritto

**Affiliations:** 1 Key Laboratory on Forest Ecology and Environmental Sciences of State Forestry Administration, Institute of Forest Ecology, Environment and Protection, Chinese Academy of Forestry, Beijing, China; 2 Co-Innovation Center for Sustainable Forestry in Southern China, Nanjing Forestry University, Nanjing, China; 3 Tree and Timber Institute, National Research Council of Italy Sesto, Fiorentino, Italy; Universidade Federal de Viçosa, BRAZIL

## Abstract

Variation in photosynthetic-nitrogen use efficiency (PNUE) is generally affected by several factors such as leaf nitrogen allocation and leaf diffusional conductances to CO_2_, although it is still unclear which factors significantly affect PNUE in tropical montane rain forest trees. In this study, comparison of PNUE, photosynthetic capacity, leaf nitrogen allocation, and diffusional conductances to CO_2_ between five Fagaceae tree species and five Leguminosae tree species were analyzed in Jianfengling tropical montane rain forest, Hainan Island, China. The result showed that PNUE of Fagaceae was significantly higher than that of Leguminosae (+35.5%), attributed to lower leaf nitrogen content per area (*N*_area_, –29.4%). The difference in nitrogen allocation was the main biochemical factor that influenced interspecific variation in PNUE of these tree species. Fagaceae species allocated a higher fraction of leaf nitrogen to the photosynthetic apparatus (*P*_P_, +43.8%), especially to Rubisco (*P*_R_, +50.0%) and bioenergetics (*P*_B_ +33.3%) in comparison with Leguminosae species. Leaf mass per area (LMA) of Leguminosae species was lower than that of Fagaceae species (-15.4%). While there was no significant difference shown for mesophyll conductance (*g*_m_), Fagaceae tree species may have greater chloroplast to total leaf surface area ratios and that offset the action of thicker cell walls on *g*_m_. Furthermore, weak negative relationship between nitrogen allocation in cell walls and in Rubisco was found for *Castanopsis hystrix*, *Cyclobalanopsis phanera* and *Cy*. *patelliformis*, which might imply that nitrogen in the leaves was insufficient for both Rubisco and cell walls. In summary, our study concluded that higher PNUE might contribute to the dominance of most Fagaceae tree species in Jianfengling tropical montane rain forest.

## Introduction

Nitrogen is one of the most important biological elements for plants, and is essential for amino acids, proteins, genetic materials, pigments, and other key organic molecules [1, 2]. Globally, nitrogen has been proposed as a critical component for photosynthesis, and leaf nitrogen content per area (*N*_area_) has a significant positive correlation with light-saturated net CO_2_ assimilation rate (*A*_max_’) [3]. Field and Mooney showed that up to 75% of leaf nitrogen was present in the chloroplasts, and within them, most of the nitrogen was allocated to the photosynthetic apparatus [4]. Therefore, photosynthetic-nitrogen use efficiency (PNUE), defined as the ratio of *A*_max_’ to *N*_area_, has been considered an important leaf trait that characterizes species in relation to their leaf economics, physiology, and survival strategy [5]. Since nitrogen availability often limits plant growth [6, 7], species with high PNUE tend to have higher growth rates [5] and higher competitive ability in natural ecosystems [8]. Improving understanding of the inherent variation of PNUE among species is therefore of great importance [9].

Interspecific variation of PNUE can be attributed to nitrogen allocation in the photosynthetic apparatus, CO_2_ diffusion from the air to the carboxylation site, and/or specific activity of photosynthetic enzymes [4, 9]. Feng et al. found a significant positive correlation between the fractions of leaf nitrogen in the photosynthetic apparatus (*P*_P_) and PNUE in *Ageratina adenophora* [10]. Rubisco constitutes approximately 50% of photosynthetic nitrogen [11], and catalyzes the limiting step that determines photosynthetic capacity [12, 13]. A significant positive correlation between the fraction of leaf nitrogen in Rubisco (*P*_R_) and PNUE was found in *Populus cathayana* [2], *Spartina alterniflora* [14], and 26 temperate plants [15]. The cell wall is of critical importance for maintaining cell shape, providing mechanical strength to withstand turgor pressure, and influencing the toughness of leaves [7, 16]. Previous research has shown that trade-offs may occur when nitrogen is allocated to cell walls versus Rubisco; thus, nitrogen in cell walls could lead to variation of PNUE [7, 17]. However, previous studies suggest that these trade-offs might only be intraspecific [15], and might exist only in species that lack nitrogen in leaves [14, 18].

The carboxylation capacity of Rubisco is dependent on CO_2_ partial pressure, since Rubisco activity is induced by chloroplastic CO_2_ [19]. Broeckx et al. found a significant positive correlation between mesophyll conductance (*g*_m_) and PNUE in six poplar (*Populus*) genotypes [20]. Xu et al. found a significant negative correlation between *C*_i_ (intercellular CO_2_ concentration)-*C*_c_ (CO_2_ concentration at carboxylation site) and PNUE in *Populus cathayana* [21]. Leaf mass per area (LMA) can be expressed as the product of leaf thickness and leaf density, and has been shown to be correlated with leaf toughness [5]. Lower LMA usually led to a higher *g*_m_ [22–24], and LMA was negatively correlated with PNUE in many species [5], however, conclusions were variable [20, 25, 26]. Furthermore, research on PNUE and influencing factors is lacking for tropical areas [27, 28].

Tropical forests account for about half of the worldwide forest cover and play an extremely vital role in global carbon fixing and cycling [29]. Despite such prominence, the factors influencing PNUE in tropical forests are still unclear [30]. Tropical forests are preference hotspots for Fagaceae and Leguminosae tree species [27, 31], which also can be found in Jianfengling tropical montane rain forest, Hainan Island, China [32]. According to Xu et al, most of the Fagaceae species were common in this area and dominated the canopy layer, especially in the primary forest [31]. In contrast, tree species in Leguminosae, which usually have nitrogen-fixation ability [27], living at the lower canopy layer, are rare and play an important role in maintaining biodiversity in Jianfengling tropical rain forest. Recent studies found that Leguminosae tree species with higher *N*_area_ did not have higher *A*_max_’ than other species [33, 34], although Wright et al. found that species with high *N*_area_ usually had high *A*_max_’, according to the worldwide leaf economic spectrum [3]. These opposite results may imply that nitrogen-fixing species may use a different strategy to utilize nitrogen as compared to non-nitrogen-fixing species. One possible explanation was that tree species in Leguminosae may allocate less nitrogen to Rubisco and bioenergetics than non-legumes, which has been proven by previous studies [33, 34]. However, these studies did not consider that *g*_m_ could also influence PNUE [35].

In this study, PNUE and influencing factors such as photosynthetic capacity, leaf nitrogen allocation, and diffusional conductances to CO_2_ in Fagaceae and Leguminosae tree species were investigated in Jianfengling tropical montane rain forest. Our aims were (1) to examine how Fagaceae and Leguminosae tree species vary in PNUE, leaf nitrogen allocation, and diffusional conductances to CO_2_; and (2) to test the relationship between Rubisco nitrogen and cell wall nitrogen in Fagaceae and Leguminosae tree species.

## Materials and methods

### Study area and plant material

This experiment was conducted in Jianfengling tropical montane rain forest (108°47′–109°02′E, 18°38′–18°48′N) in Hainan Province. This area belongs to the low latitude tropical island monsoon climate region, which exhibits distinct dry and wet seasons. Mean annual precipitation is 2449 mm, occurring mainly from May to October. The average annual temperature is 19.8°C, the average monthly minimum and maximum temperatures are 14.8°C and 23.3°C, and the active accumulated temperature above 10°C is 7200°C [36, 37]. Tropical montane rain forests are distributed across rolling topography, with rich plant species and a complex community structure, containing more than 280 tree and shrub species within 62 families [38]. The soil type is yellow soil, or yellow brick soil, with a high concentration of soil water and humus [39].

The study site was located in a tropical montane rain forest ranging from 890 to 930 m above sea level. Five Fagaceae tree species (*Lithocarpus fenzelianus*, *Castanopsis hystrix*, *Ca*. *fissa*, *Cyclobalanopsis phanera*, *Cy*. *patelliformis*), and five Leguminosae tree species (*Ormosia fordiana*, *O*. *semicastrata*, *O*. *balansae*, *Pithecellobium clypearia*, *P*. *lucidum*) were chosen for this study. Trees of these two families were late species except *Ca*. *hystrix*, but *Ca*. *hystrix* could live a long time (up to 400 years), thus these species could co-occurring for a long period. Five healthy and similar sized mature trees per species were chosen. On sunny days from 9:00 am to 11:00 am in July and August of 2015, five to seven 1- to 2-m-long healthy annual branches that were exposed to the sun were cut from the top of each objective tree. The best shoot was chosen and rapidly put into a bucket of fresh water after cutting the bottom to prevent gas embolism. One healthy leaf per shoot was chosen for the determination of gas exchange parameters [28, 40, 41].

### Determination of gas exchange measurements

Gas exchange parameters were determined with a LiCor-6400 portable photosynthesis system (LI-COR, Lincoln Nebraska, USA). Photosynthetic response to photosynthetic photon flux density (PPFD) and *C*_i_ were determined on one healthy leaf per shoot. Under 380 μmol mol^–1^ of leaf chamber CO_2_ concentration, photosynthetic rates were measured at photon flux densities of 1500, 1200, 1000, 800, 600, 400, 200, 150, 100, 80, 50, 30, 20, 10 and 0 μmol·m^–2^·s^–1^. Under saturated PPFD, photosynthetic rates were detected using the same leaf at leaf chamber CO_2_ concentrations of 380, 200, 150, 100, 80, 50, 380, 600, 800, 1000, 1200, 1500, 1800 and 2000 μmol mol^–1^ [20]. Relative humidity of the air in the leaf chamber was maintained at 60–70%, and leaf temperature was set at 30°C. The photosynthetic rate and intercellular CO_2_ concentration of each sampled leaf were recorded ten times after 200 s under each PPFD and CO_2_ step.

Light-saturated net CO_2_ assimilation rate was measured under saturated PPFD and leaf chamber CO_2_ concentration of 380 μmol mol^–1^. Dark respiration (*R*_n_) was measured under leaf chamber CO_2_ concentrations of 380 μmol mol^–1^ and a photon flux density of 0 μmol m^–2^ s^–1^, and light-saturated day respiration rate (*R*_d_) was determined as half of the *R*_n_ value [42]. Light- and CO_2_-saturated net CO_2_ assimilation rate (*A*_max_) was calculated according to Farquhar et al. [12].

### Determination of chlorophyll fluorescence and mesophyll conductance

Fluorescence yield was measured with a LiCor–6400 leaf chamber fluorometer (6400–40, LI-COR, Lincoln Nebraska, USA) using the same leaf. Chamber relative humidity and leaf temperature were controlled under the same conditions as those of the gas exchange parameters. Leaf chamber CO_2_ concentration was set to 380 μmol mol^–1^. Before measurement, each leaf sample was illuminated with a saturating level of PPFD provided by the LiCor LED light source for 5–20 min to achieve fully photosynthetic induction. Intensity, rate, filter, and gain were set at <1 μmol m^–2^ s^–1^, 20 kHz, 1 Hz, and 10 times, respectively, to measure fluorescence yield (⊿*F*/*F*_m_′). Then constant values of each leaf sample were recorded 10 times after 200 s [43]. The photosynthetic electron transport rate (*J*_f_) was calculated based on Loreto et al. [44]:

$$J_{f}=PPFD\times \frac{∆F}{F_{m}\prime }\times Leafreflu\times PARDistPhotosys$$

*PPFD* is the photosynthetic photon flux density; *Leafreflu* is leaf absorptance, valued between 0.82–0.85 [45], we use 0.85 in this paper; *PARDistPhotosys* is the fraction of quanta absorbed by photosystem II, valued 0.5 [44]. The mesophyll conductance (*g*_m_) was calculated using the variable *J* method described by Harley et al. [46–50]:

$$g_{m}=\frac{A_{max}’}{C_{i}-\{\frac{\Gamma^{*}[J_{f}+8(A_{max}’+R_{d})]}{J_{f}-4(A_{max}’+R_{d})}\}}$$

Where *R*_d_, *C*_i_ and *A*_max_’ were determined from gas exchange parameters. The *g*_m_ value computed for *A*_max_’ was obtained for light-saturated and *C*_i_ of 150–350 μmol mol^–1^. Over this *C*_i_ range, the *g*_m_ value was stable, and the estimates of *g*_m_ were relatively insensitive to minor errors in Γ*, *R*_d_, and *A*_max_’ [42, 43, 51].

Two other methods which were described by Ethier and Gu also used to calculate *g*_m_. Ethier and Livingston [52]present an alternative *A*-*C*_i_ curve fitting method that accounts for *g*_m_ through a non-rectangular hyperbola version of the model of Farquhar et al. [12], and Sharkey et al. [53] had developed an Excel spreadsheet to estimate *g*_m_ and other parameters based on this method. The EDO method described by Gu et al. [54] could estimate up to eight parameters including *g*_m_, therefore, our data was uploaded in the LeafWeb server (http://www.leafweb.org/) in order to have an automated analysis of *A*-*C*_i_ curves.(*g*_m_ estimated by three methods see S1 Table).

### Determination of *V*_cmax_ and *J*_max_

There was no significant difference between *g*_m_ calculated by three methods; therefore, we use a mean value of *g*_m_ to calculate *C*_c_:

$$C_{C}=C_{i}-\frac{A_{max}’}{g_{m}}$$

*C*_c_ was used to fit *A*-*C*_c_ curve, then maximum carboxylation rate (*V*_cmax_) were calculated according to Farquhar et al. [12], and the maximum electron transport rate (*J*_max_) was calculated according to Loustau et al. [55]. The fitting model was run using the *in vivo* Rubisco kinetics parameters (i.e. *K*_o_, *K*_c_, and their activation energy) measured by Niinemets and Tenhunen [13]. The CO_2_ photo compensation point (Γ*) value was 54.76 at 30°C, according to Bernacchi et al [56].

### Determination of additional leaf traits

After determination of the gas exchange parameters and fluorescence yield, leaf samples and nearby leaves (30–50 leaves in total per shoot), were taken from each shoot. The surface area of 10–20 leaves was measured by scanner (Perfection v700 Photo, Epson, Nagano-ken, Japan). Leaves were subsequently oven-dried at 80°C for 48 h to constant weight, dry weight was measured using an analytic balance, and then LMA was calculated. Dried leaf samples were ground into a dry flour, nitrogen concentration was determined by a VELP automatic Kjeldahl nitrogen determination apparatus (UDK-139, Milano, Italy), and then leaf nitrogen per mass (*N*_mass_) and leaf nitrogen per area (*N*_area_) were calculated.

The remaining 20–30 leaves were frozen and returned for laboratory analysis. One gram of frozen leaves (5–10 leaves) were cut into small pieces and weighed into 5–10 mg samples. Absolute chlorophyll concentration measurements were conducted using 95% (v/v) alcohol extracts of leaf tissue and a Shimadzu visible-ultraviolet spectrophotometer (UV 2250, Fukuoka, Japan), chlorophyll concentration see S2 Table. The remaining frozen leaves were used to determine cell wall nitrogen content according to Onoda et al. [7]. The fraction of leaf nitrogen allocated to cell walls (*P*_CW_) represents the ratio of cell wall nitrogen content to total nitrogen content.

### Calculation of nitrogen allocation in the photosynthetic apparatus

Nitrogen allocation fractions of each component in the photosynthetic apparatus were calculated according to Niinemets and Tenhunen [13]; this method has been widely used in recent years [2, 57, 58].

$$P_{R}=\frac{V_{cmax}}{6.25\times V_{cr}\times LMA\times N_{mass}}$$

$$P_{B}=\frac{J_{max}}{8.06\times J_{mc}\times LMA\times N_{mass}}$$

$$P_{L}=\frac{C_{Chl}}{C_{B}\times N_{mass}}$$

Where *C*_Chl_ was the chlorophyll concentration (mmol g^–1^), *V*_cr_ was the specific activity of Rubisco (μmol CO_2_ g^–1^ Rubisco s^–1^), *J*_mc_ was the potential rate of photosynthetic electron transport (μmol electrons μmol^–1^ Cyt f s^–1^), and *C*_B_ was the ratio of leaf chlorophyll to leaf nitrogen during light-harvesting (mmol Chl (g N)^–1^). *V*_cr_, *J*_mc_, and *C*_B_ were calculated according to Niinemets and Tenhunen [13]. *P*_R_, *P*_B_, and *P*_L_ were the fraction of leaf nitrogen allocated to Rubisco, bioenergetics, and the light-harvesting components (g g^–1^), respectively. The leaf nitrogen allocated to the photosynthetic apparatus (*P*_P_) was calculated as the sum of *P*_R_, *P*_B_, and *P*_L_.

### Calculation of sensitivity

To test the importance of each factor in altering PNUE, all factors which might influence PNUE were used to construct a multi-linear regression model. The value of each factor for the average of the Leguminosae species was replaced with the value for the Fagaceae species This enabled us to assess the proportion of the total difference in PNUE between the two families, attributable to each factor [59].

### Statistical analysis

Differences between species and families were analyzed by one-way analysis of variance (ANOVA), and a post hoc test (LSD test) was conducted if the differences were significant. The significance of the correlation between each pair of variables was tested with a Pearson correlation (two-tailed). Regression analyses of *N*_area_ with *A*_max_’ and *P*_P_, *P*_L_, *P*_R_, *P*_B_ with PNUE used one-way ANCOVA to determine correlations between variables and subsequent differences in those correlations between Fagaceae and Leguminosae tree species. All analyses were carried out using Statistical Product and Service Solutions 17.0 (SPSS17.0, Chicago, USA).

## Results

**Fig 1:**
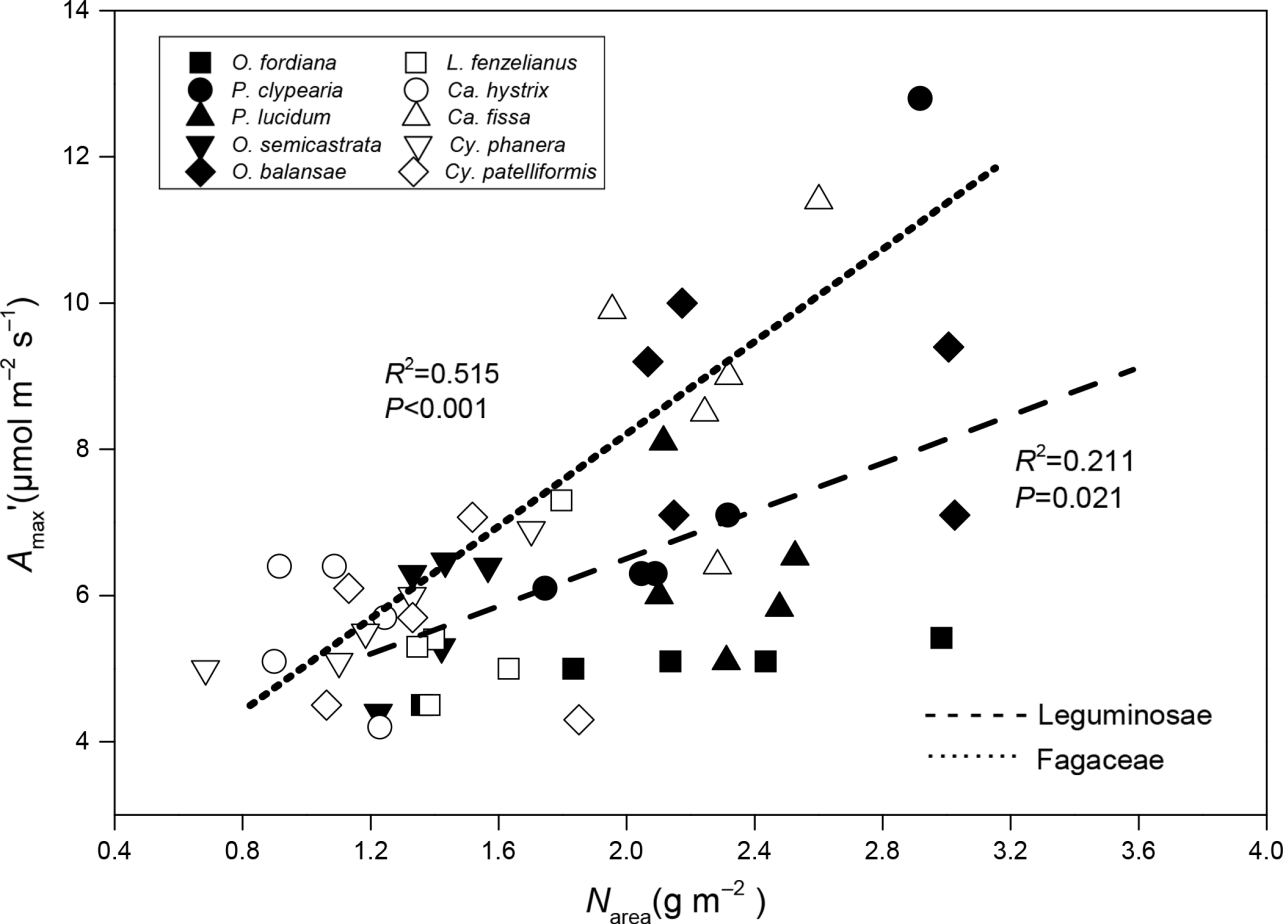
Regression analysis of leaf nitrogen content per area (*N*_area_) with light-saturated net CO_2_ assimilation rate (*A*_max_’) in 10 Jianfengling tree species leaves. The determination coefficient (*R*^2^) and *P*-value are shown. The lines fitted separately for Leguminosae and Fagaceae families are significantly different (*P*<0.05) according to the result of a one-way ANCOVA with *A*_max_’ as a dependent variable, families as fixed factors, and *N*_area_ as a covariate.

The differences among the 10 studied species were significant in all variables except *C*_i_, and *P*_L_, in which the differences were not significant (Tables 1–4). *N*_area_ and *A*_max_ of Leguminosae species were significantly higher than those of Fagaceae species (+41.6% and +22.7%, respectively). In contrast, PNUE, LMA and *C*_c_ of Leguminosae species were 26.2%, 15.4%, and 15.88% lower, respectively, than those of Fagaceae species. No significant differences were found in *g*_s_, *g*_m_, *A*_max_’ *C*_i_ *V*_cmax_ or *J*_max_ between families (Tables 1–3). *N*_area_ had a significant positive correlation with *A*_max_’ in Fagaceae and Leguminosae tree species leaves, but Fagaceae species showed significantly higher *A*_max_’ than Leguminosae species at the same value of *N*_area_ (Fig 1).

**Table 1:** Light-saturated photosynthesis (*A*_max_’), leaf nitrogen content per area (*N*_area_), leaf mass per area (LMA) and photosynthetic-nitrogen use efficiency (PNUE) in 10 Jianfengling tree species leaves.

Species	Families	*A*_max_’(μmol m^–2^ s^–1^)	*N*_area_ (g m^–2^)	LMA(g m^–2^)	PNUE (μmol mol^–1^ s^–1^)
*O*. *fordiana*	Leguminosae	5.02±0.15^cd^	2.15±0.27^a^	82.26±8.86^cde^	34.58±3.64^fgh^
*P*. *clypearia*		7.72±1.28^abc^	2.22±0.20^a^	102.72±7.76^bcd^	47.74±3.64^cdefgh^
*P*. *lucidum*		6.31±0.50^bcd^	2.31±0.09^a^	70.25±4.96^de^	38.78±4.06^efgh^
*O*. *semicastrata*		5.77±0.40^cd^	1.40±0.06^b^	74.71±2.53^de^	57.82±3.08^bcdef^
*O*. *balansae*		8.56±0.61^ab^	2.48±0.22^a^	117.70±12.65^bc^	49.98±5.88^cdefgh^
*L*. *fenzelianus*	Fagaceae	5.50±0.48^cd^	1.51±0.09^b^	138.91±6.08^a^	50.96±2.80^cdefg^
*Ca*. *hystrix*		5.56±0.42^cd^	1.07±0.07^b^	85.28±2.21^cde^	74.34±8.54^abc^
*Ca*. *fissa*		9.04±0.82^ab^	2.28±0.10^a^	117.41±4.21^bc^	55.72±5.18^bcdef^
*Cy*. *phanera*		5.70±0.34^cd^	1.20±0.16^b^	89.44±10.89^cde^	70.42±8.12^abcd^
*Cy*. *patelliformis*		5.53±0.51^cd^	1.38±0.14^b^	97.86±3.21^bcd^	58.52±7.14^abcdef^
*F*		5.216***	11.732***	9.123***	4.999***
	Leguminosae	6.68±0.39^a^	2.11±0.11^a^	89.53±4.94^b^	45.78±2.38^b^
	Fagaceae	6.27±0.36^a^	1.49±0.10^b^	105.79±3.59^a^	62.02±3.22^a^
	*F*	0.602	18.104***	5.591*	15.953***

Mean values (± SE) are shown (n = 5 for species and n = 25 for families). Different letters indicate significant differences between species and families (ANOVA, LSD test, *P* < 0.05).

*F*-ratios with statistically significant values are denoted by * *P*<0.05, ** *P*<0.01, *** *P*<0.001.

**Table 2:** Stomatal conductance (*g*_s_), mesophyll conductance (*g*_m_), intercellular CO_2_ concentration (*C*_i_), CO_2_ concentration at carboxylation site (*C*_c_) in 10 Jianfengling tree species leaves.

Species	Families	*g*_s_(molCO_2_m^–2^ s^–1^)	*C*_i_(μmol mol^–1^)	*g*_m_(molCO_2_ m^–2^ s^–1^)	*C*_c_(μmol mol^–1^)
*O*. *fordiana*	Leguminosae	0.052±0.007^cdef^	289.44±7.57^abcd^	0.027±0.001^efg^	107.61±6.52^bcde^
*P*. *clypearia*		0.061±0.017^bcdef^	277.23±16.83^abcde^	0.036±0.005^cdefg^	81.19±19.65^cdef^
*P*. *lucidum*		0.053±0.016^cdef^	248.46±12.84^cde^	0.047±0.005^bcde^	105.26±9.36^bcde^
*O*. *semicastrata*		0.040±0.007^def^	263.51±5.77^bcde^	0.027±0.001^efg^	67.49±14.41^def^
*O*. *balansae*		0.101±0.005^abc^	282.81±7.50^abcd^	0.059±0.008^bc^	124.66±10.42^abcd^
*L*. *fenzelianus*	Fagaceae	0.082±0.025^bcde^	298.60±21.05^abc^	0.029±0.003^defg^	114.69±11.51^abcd^
*Ca*. *hystrix*		0.043±0.002^def^	266.48±7.07^abcde^	0.031±0.002^defg^	93.71±5.58^bcdef^
*Ca*. *fissa*		0.089±0.009^abcd^	256.22±8.63^bcde^	0.091±0.011^a^	141.78±8.83^ab^
*Cy*. *phanera*		0.044±0.005^def^	256.29±11.36^bcde^	0.043±0.004^cdef^	124.29±7.89^abcd^
*Cy*. *patelliformis*		0.047±0.003^ef^	263.81±11.05^bcde^	0.036±0.005^cdefg^	102.07±9.84^bcde^
*F*		3.692**	1.847	13.983***	3.880**
	Leguminosae	0.062±0.006^a^	272.26±5.37^a^	0.040±0.003^a^	97.00±6.99^b^
	Fagaceae	0.061±0.006^a^	268.24±6.13^a^	0.046±0.005^a^	115.31±5.02^a^
	*F*	0.009	0.243	1.036	4.583*

*g*_m_ was the mean value of three methods (Harley, Ethier and Gu), respective value of *g*_m_ calculated by three methods see S1 Table. Mean values (± SE) are shown (n = 5 for species and n = 25 for families). The meaning of the letter in the same column and the definition of statistical significance have been described in Table 1; data were measured in light-saturated and atmospheric CO_2_ concentrations of 380 μmol mol^–1^.

**Table 3:** Light- and CO_2_-saturated net CO_2_ assimilation rate (*A*_max_), maximum carboxylation rate (*V*_cmax_), and maximum electron transport rate (*J*_max_) in 10 Jianfengling tree species leaves.

Species	Families	*A*_max_(μmol m^–2^ s^–1^)	*V*_cmax_(μmol m^–2^ s^–1^)	*J*_max_(μmol m^–2^ s^–1^)
*O*. *fordiana*	Leguminosae	13.51±1.42^bcde^	36.40±2.13^cd^	67.78±3.43^bcdefg^
*P*. *clypearia*		14.44±0.73^abcde^	37.83±2.50^cd^	73.90±4.91^bcdef^
*P*. *lucidum*		18.60±2.77^abc^	42.90±4.29^bcd^	81.61±5.57^bcd^
*O*. *semicastrata*		11.89±0.77^cde^	36.62±1.41^cd^	64.40±4.23^cdefg^
*O*. *balansae*		17.46±1.81^abcd^	41.98±3.30^cd^	78.48±8.92^bcde^
*L*. *fenzelianus*	Fagaceae	12.76±0.80^cde^	40.08±4.76^cd^	63.43±2.65^defg^
*Ca*. *hystrix*		10.47±1.44^cde^	36.85±1.70^cd^	61.08±5.16^defg^
*Ca*. *fissa*		17.25±0.57^abcd^	60.45±3.16^a^	98.38±2.96^a^
*Cy*. *Phanera*		10.69±2.06^cde^	48.10±5.72^bc^	79.89±6.27^bcde^
*Cy*. *patelliformis*		10.71±1.03^cde^	35.74±1.47^cd^	57.86±3.37^defg^
*F*		4.154**	9.184***	5.887***
	Leguminosae	15.18±0.85^a^	39.17±1.32^a^	73.30±2.69^a^
	Fagaceae	12.37±0.75^b^	43.85±2.92^a^	72.13±3.56^a^
	*F*	6.127*	2.689	0.068

Mean values (± SE) are shown (n = 5 for species and n = 25 for families). The meaning of the letter in the same column and the definition of statistical significance have been described in Table 1.

**Table 4:** Fraction of leaf nitrogen allocated to Rubisco (*P*_R_), bioenergetics (*P*_B_), light-harvesting components (*P*_L_), photosynthetic apparatus (*P*_P_), cell wall (*P*_CW_), and other parts (1-*P*_P_-*P*_CW_, *P*_Other_) in 10 Jianfengling tree species leaves.

Species	Families	*P*_R_(g g^–1^)	*P*_B_(g g^–1^)	*P*_L_(g g^–1^)	*P*_P_(g g^–1^)	*P*_CW_(g g^–1^)	*P*_Other_(g g^–1^)
*O*. *fordiana*	Leguminosae	0.099±0.012	0.024±0.003	0.013±0.003^bc^	0.14±0.017	0.24±0.015^de^	0.63±0.018^bcd^
*P*. *clypearia*		0.12±0.012	0.026±0.002	0.015±0.001^abc^	0.16±0.014	0.15±0.008^f^	0.70±0.015^abc^
*P*. *lucidum*		0.12±0.018	0.027±0.003	0.016±0.001^abc^	0.16±0.022	0.09±0.003^g^	0.75±0.020^ab^
*O*. *semicastrata*		0.16±0.009	0.034±0.002	0.018±0.001^abc^	0.21±0.009	0.23±0.009^de^	0.56±0.017^cde^
*O*. *balansae*		0.12±0.012	0.025±0.004	0.016±0.001^abc^	0.17±0.016	0.30±0.008^cd^	0.54±0.018^de^
*L*. *fenzelianus*	Fagaceae	0.15±0.006	0.031±0.001	0.015±0.001^abc^	0.20±0.007	0.28±0.016^cde^	0.53±0.021^de^
*Ca*. *hystrix*		0.20±0.014	0.041±0.003	0.014±0.002^bc^	0.26±0.017	0.46±0.030^a^	0.28±0.022^g^
*Ca*. *fissa*		0.14±0.012	0.029±0.002	0.016±0.002^abc^	0.19±0.013	0.13±0.006^f^	0.68±0.010^abc^
*Cy*. *phanera*		0.20±0.035	0.047±0.007	0.017±0.002^abc^	0.27±0.041	0.29±0.025^cd^	0.44±0.053^f^
*Cy*. *patelliformis*		0.19±0.024	0.034±0.005	0.018±0.001^ab^	0.24±0.028	0.37±0.030^b^	0.39±0.053^f^
*F*		4.931***	4.812***	1.038	4.926***	40.559***	26.778***
	Leguminosae	0.12±0.006^b^	0.027±0.001^b^	0.015±0.001^a^	0.16±0.008^b^	0.200±0.016^b^	0.63±0.018^a^
	Fagaceae	0.18±0.010^a^	0.036±0.002^a^	0.016±0.001^a^	0.23±0.012^a^	0.301±0.024^a^	0.46±0.031^b^
	*F*	22.217***	14.299***	0.098	20.691***	13.522**	22.645***

Mean values (± SE) are shown (n = 5 for species and n = 25 for families). The meaning of the letter in the same column and the definition of statistical significance have been described in Table 1.

The fraction of leaf nitrogen allocated to other parts (*P*_Other_, 1-*P*_P_-*P*_CW_) was the highest both in Fagaceae and Leguminosae tree species leaves, followed by *P*_CW_ and *P*_P_. *P*_Other_ of Leguminosae was significantly higher than that of Fagaceae (+37.0%), yet *P*_P_ and *P*_CW_ were significantly lower than that of Fagaceae (–30.4% and –33.6%, respectively). *P*_R_ was the highest both in Fagaceae and Leguminosae tree species leaves, followed by *P*_B_ and *P*_L_. *P*_R_ and *P*_B_ in Fagaceae were significantly higher than those in Leguminosae (+50.0% and +33.3%, respectively), but there was no significant difference found in *P*_L_ between Fagaceae and Leguminosae (Table 4).

**Fig 2:**
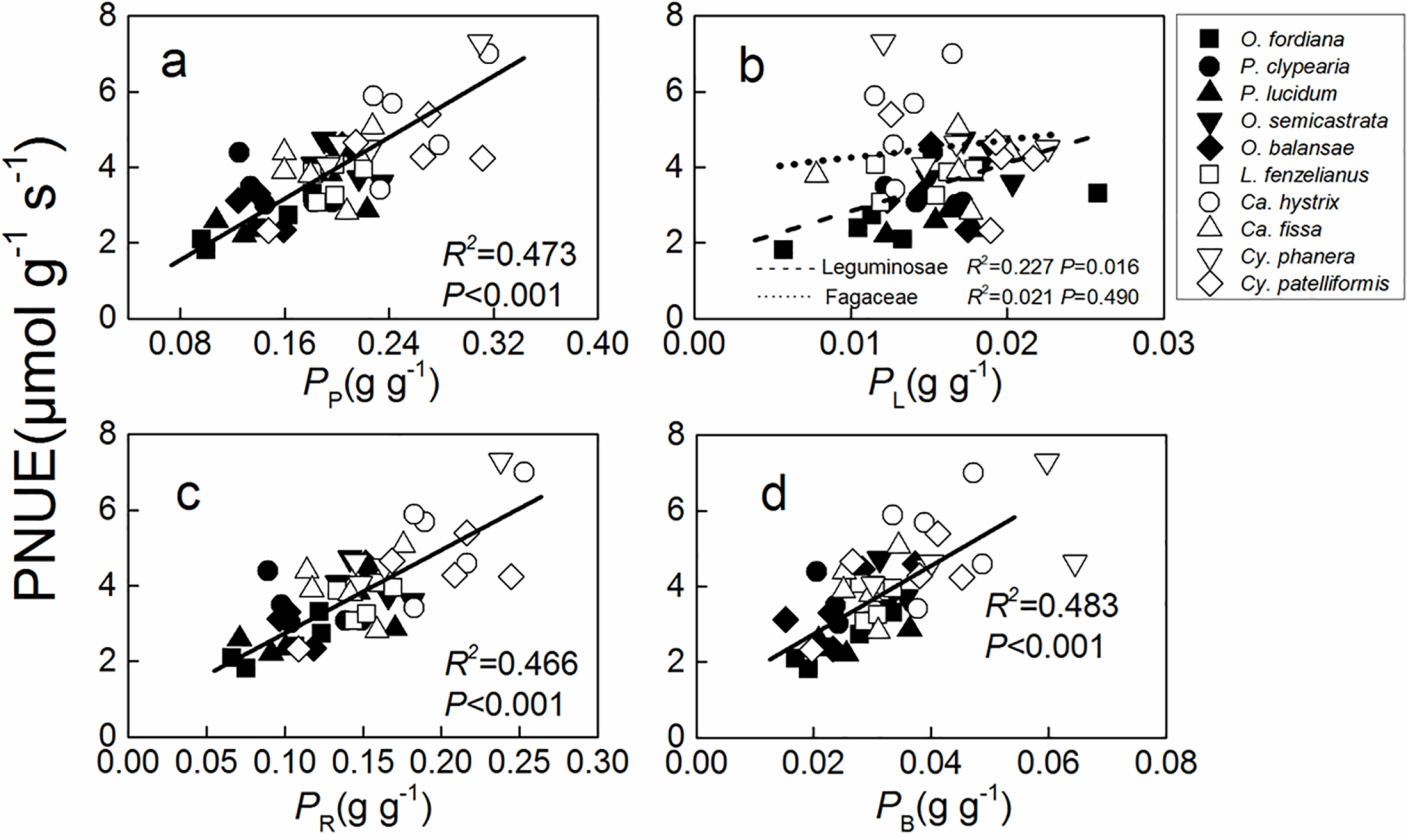
**Regression analysis of the fraction of leaf nitrogen allocated to (a) the photosynthetic apparatus (*P***_**P**_**), (b) light-harvesting components (*P***_**L**_**), (c) Rubisco (*P***_**R**_**), and (d) bioenergetics (*P***_**B**_**) with photosynthetic-nitrogen use efficiency (PNUE) in 10 Jianfengling tree species leaves.** The determination coefficient (*R*^2^) and *P*-value are shown. The lines fitted separately for Leguminosae and Fagaceae families are significantly different in plots **b** (*P*<0.05) according to the result of a one-way ANCOVA with PNUE as a dependent variable, families as fixed factors, and *P*_L_ as a covariate.

**Fig 3:**
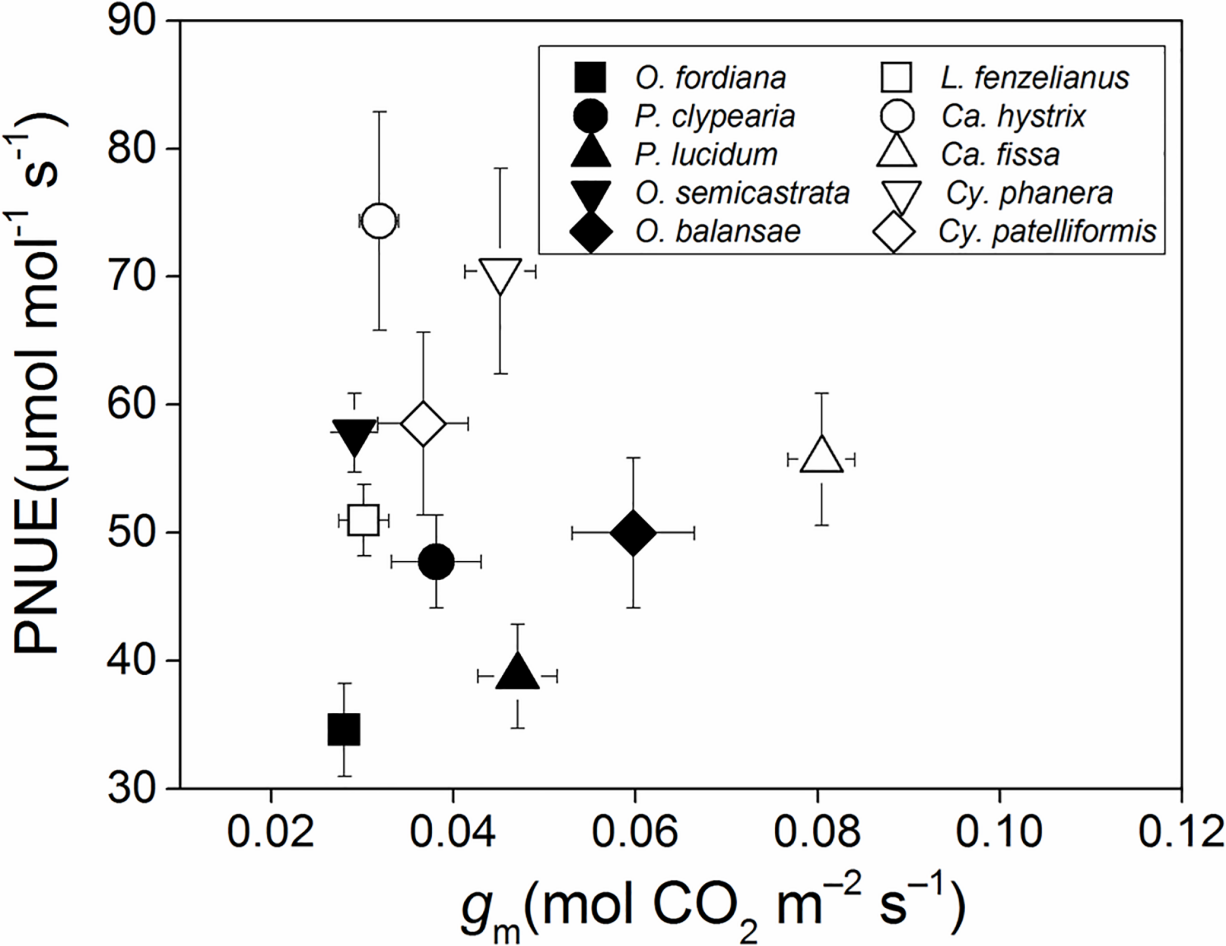
Mesophyll conductance (*g*_m_) in relation to photosynthetic-nitrogen use efficiency (PNUE) in 10 Jianfengling tree species leaves. There was no significant difference between *g*_m_ calculated by three methods, we use the mean value of three *g*_m_ (Harley, Ethier and Gu).

**Fig 4:**
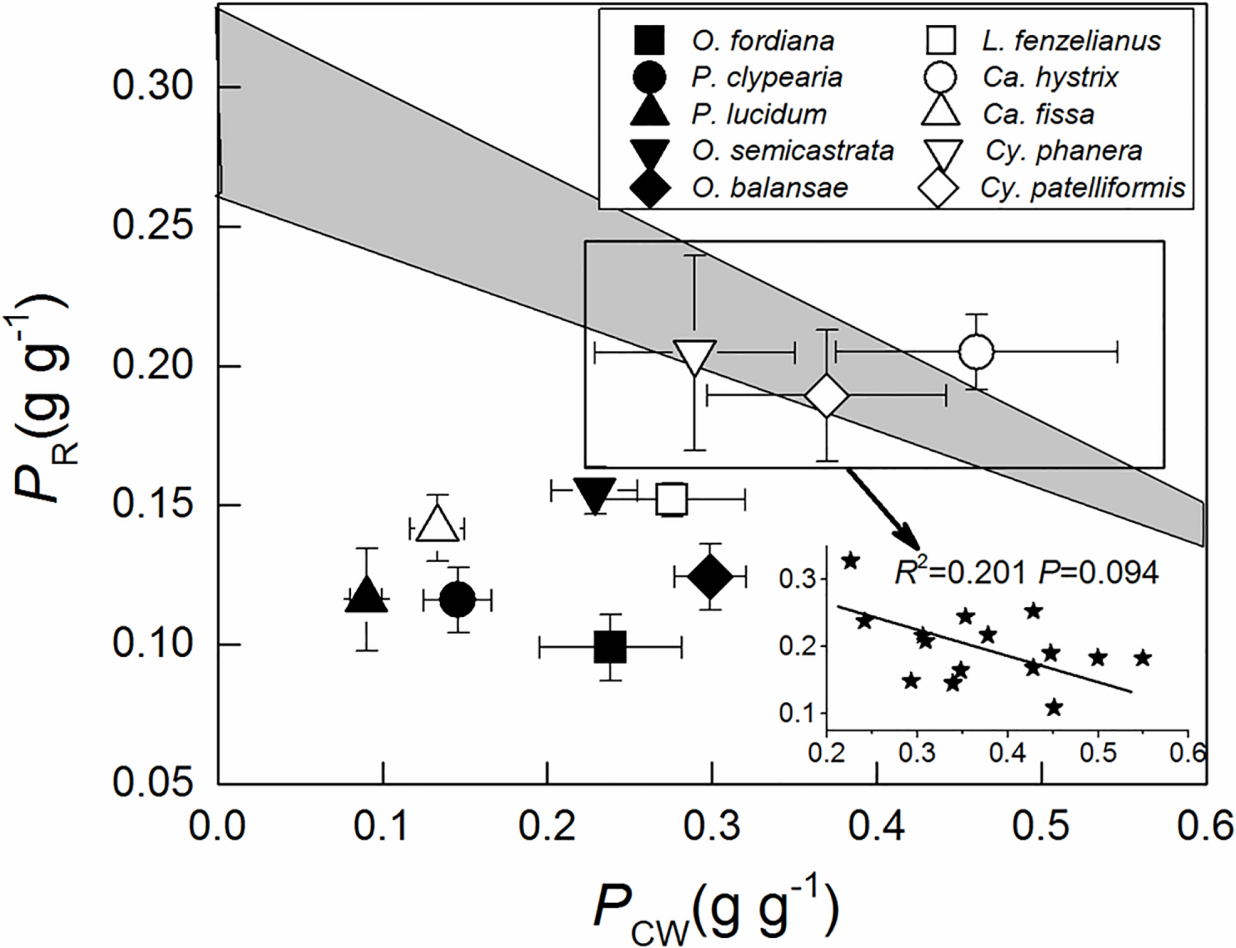
Fraction of leaf nitrogen allocated to the cell wall (*P*_CW_) in relation to the fraction of leaf nitrogen allocated to Rubisco (*P*_R_) in 10 Jianfengling tree species leaves. The shaded zone represents the distribution area of *P*_CW_ and *P*_R_ when trade-off existed [18].

*P*_P_, *P*_R_, and *P*_B _had a significant positive correlation with PNUE in the studied tree species (*R*^2^≥0.466), and *P*_L_ had a significant positive correlation with PNUE in Leguminosae tree species, but no significant correlation was found in Fagaceae tree species (Fig 2). Mesophyll conductance of Fagaceae and Leguminosae tree species was not significantly related to the PNUE (Fig 3). Weak negative relationship was between nitrogen allocation in cell walls and in Rubisco for *Castanopsis hystrix*, *Cyclobalanopsis phanera* and *Cy*. *patelliformis*. (Fig 4).

Replacing *P*_B_ of Leguminosae species with that of Fagaceae made the highest proportion of changes to PNUE in Leguminosae (-21.74%), followed by *P*_R_ (+11.95%) and *N*_area_ (+6.63%). Replacing *C*_i_ *g*_m_ *J*_max_ *P*_CW_ and *P*_Other_ of Leguminosae species with those of Fagaceae made almost no change to PNUE in Leguminosae (≤±2%, Table 5).

**Table 5:** The average PNUE and related factors for Leguminosae and Fagaceae tree species, and a sensitivity analysis to assess the relative importance of each of these factors in explaining the difference in PNUE.

Families		PNUE	*N*_area_	*A*_max_	*g*_s_	*C*_i_	*g*_m_	*C*_c_	LMA	*A*_max_*‘*
Leguminosae	Mean	45.78	2.11	6.68	0.062	272.26	0.040	97.00	89.53	89.53
Fagaceae	Mean	62.02	1.49	6.27	0.061	268.24	0.046	115.31	105.79	105.79
	PNUE expl (%)		*-6*.*63%*	*5*.*33%*	*2*.*01%*	*1*.*33%*	*1*.*24%*	*2*.*11%*	*4*.*51%*	*2*.*52%*
**Families**		**PNUE**	***V***_**cmax**_	***J***_**max**_	***P***_**R**_	***P***_**B**_	***P***_**L**_	***P***_**P**_	***P***_**CW**_	***P***_**Other**_
Leguminosae	Mean	45.78	39.17	73.3	0.12	0.027	0.015	0.16	0.2	0.63
Fagaceae	Mean	62.02	46.85	72.13	0.18	0.036	0.016	0.23	0.301	0.46
	PNUE expl (%)		*-2*.*74%*	*0*.*38%*	*11*.*95%*	*21*.*47%*	*2*.*51%*	*-5*.*34%*	*1*.*77%*	*-1*.*12%*

PNUE expl (%) stands for the percentage of the difference in PNUE between Leguminosae and Fagaceae tree species explained by substituting a given factor of the Leguminosae species with the value for the Fagaceae species [59]. Values for all important factors are printed in italics, with the most important factor being underlined. A positive value means that changing the factor resulted in a PNUE that exceeded that of Leguminosae.

## Discussion

Leguminosae tree species were significantly lower in PNUE (Table 1) than Fagaceae, which was consistent with the results of other authors who reported that legumes had lower PNUE than non-legumes [33, 34]. Fagaceae with higher PNUE may have higher growth rates than Leguminosae [5]. Most of the tree species in Fagaceae were dominant species with a total importance value that accounted for 16.06% of the sum of importance values over all species in the study area, whereas the proportion of species in Leguminosae was only 2.67% [32]. The higher PNUE of Fagaceae species might be one factor that explains their higher competitive ability in tropical montane rain forest [8, 60], although factors affecting competition among species in a community are complicated and require further exploration [61].

Leguminosae tree species had lower PNUE first attributed to their significantly higher *N*_area_ than those in Fagaceae (Table 1). These results agreed with earlier reports on two Acacia species (*Acacia auriculiformis* and *A*. *mangium*) and four Eucalyptus species (*Eucalyptus camaldulensis*, *E*. *urophylla*, *E*. *grandis*, and *E*. *globulus*) [34], and in the Leguminous *Alhagi sparsifolia* and non-leguminous *Tamarix ramosissima* and *Karelinia caspica* [33]. *N*_area_ had a significant positive correlation with *A*_max_’ according to the worldwide leaf economic spectrum [3], which was also found in our study (Fig 1) and reflects the importance of nitrogen in photosynthesis. But inversely, the Fagaceae species showed significantly higher *A*_max_’ than the Leguminosae species at the same value of *N*_area_ (Fig 1). In fact, *J*_max_ and *V*_cmax_ were not significantly different between families, except for *A*_max_, which was higher in Leguminosae than in Fagaceae species (*P*<0.05) (Table 3). These findings indicate that there were no strong differences in the biochemical parameters of photosynthetic capacity [43, 62]. These results imply that the optimization of nitrogen allocation within leaves is a key adaptive mechanism to maximize photosynthesis [61], and more important than total nitrogen.

CO_2_ conductance can affect leaf photosynthetic capacity and PNUE by affecting the supply of CO_2_ to the sites of carboxylation [20, 26]. There was no significant difference in *C*_i_, *g*_s_, or *g*_m_ between families (Table 2). Although Fagaceae had higher *C*_c_ than Leguminosae (Table 2), there was no significant difference in *V*_cmax_, which demonstrates an equal ability for using CO_2_ in the sites of carboxylation between different families (Table 3) [63, 64]. These findings indicate that there were no strong differences in CO_2_ conductance between these families. Broeckx et al. found a significant positive relationship between *g*_m_ and PNUE in six poplar (*Populus*) genotypes [20], and suggested that nitrogen involved in carbonic anhydrases and aquaporins [65] could play a role in mesophyll conductance (*g*_m_) by changing the nature of the diffusing molecule [66] and facilitating CO_2_ diffusion through membranes [67]; however, this study was only conducted on one species. Our results showed no significant relationship between *g*_m_ and PNUE in these 10 tree species (Fig 3); the result of sensitivity analysis also proved that *g*_m_ was not important in altering PNUE (-0.6%, Table 5). The LMA of these species were significantly different (*P*<0.001, Table 1), which signifies a large difference in the leaf structure of these species. Leaf structure greatly influenced *g*_m_ [68, 69], thus interspecific differences in leaf structure may weaken the correlation between PNUE and *g*_m_.

The fraction of the total leaf nitrogen allocated to the photosynthetic apparatus [10], especially to Rubisco and bioenergetics, was a key factor that influenced PNUE [2, 13–15]. In this study, *P*_P_, *P*_R_, and *P*_B_ were significantly and positively related to PNUE (Fig 2); the five Fagaceae tree species had significantly higher *P*_P_, especially *P*_R_ and *P*_B_, than the five Leguminosae tree species (Table 4), which led to their higher PNUE. These results agreed with earlier reports by Zhu et al. [33], Novriyanti et al. [34], and Feng et al. [57]. Light is also an important limited resource factor for plants in tropical montane rain forest. The ability of capturing and utilizing light for plants was an important determinant of growth potential and fitness [70]. The result of sensitivity analysis also found that *P*_B_ and *P*_R_ were more important than other factors in altering PNUE (+21.47% and +11.95%, Table 5). Poorter and Evans [59] considered *P*_P_ to be the most important factor in altering PNUE for 10 plants grown at low irradiance (200 μmol·m^–2^·s^–1^), but Rubisco specific activity was the most important for PNUE of high-light grown plants (1000 μmol·m^–2^·s^–1^). The key factors for PNUE may be influenced by interspecific difference and environmental factors.

Although there was no difference in leaf nitrogen allocated to light-harvesting components (P_*L*_) between Leguminosae and Fagaceae tree species(Table 4), Leguminosae had higher leaf nitrogen content per area (N_*area*_, Table 1), means higher nitrogen content in light-harvesting components (0.032±0.0017 g·m^–2^ vs 0.024±0.002 g·m^–2^, *P* = 0.004). Leguminous tree species also had higher *P*_L_/*P*_R_ than Fagaceae (0.288±0.026 vs 0.229±0.021, *P* = 0.021).We observed that Fagaceae tree species usually have higher tree height (upper canopy) than those in Leguminosae, which height niches distributed under canopy [31]. This living environment may encourage Leguminosae with higher nitrogen in the light-harvesting system to obtain enough light for growth [71].

Although Fagaceae tree species had higher LMA than Leguminosae, there was no significant difference between their *g*_m_ (Tables 1 and 2). Variations in LMA are often inversely correlated with *g*_m_ [22–24]. In contrast, some studies have found a positive correlation between LMA and *g*_m_ [25, 26]. Broeckx et al. found no significant correlation between LMA and *g*_m_ in 12 poplar genotypes [20]. If higher LMA is a result of mesophyll cell wall thickening, it will reduce *g*_m_ [68, 69]; if it is associated with a greater number of mesophyll layers, and accordingly, greater chloroplast to total leaf surface area ratios, it will improve *g*_m_ [72]. Fagaceae tree species showed significantly higher *P*_CW_ than Leguminosae (Table 4); this may imply a greater cell wall density and thicker cell wall [15]. Thus, Fagaceae tree species may also have greater chloroplast to total leaf surface area ratios which offset the action of the thicker cell wall on *g*_m_ [24].

Weak negative relationship was between nitrogen allocation in cell walls and in Rubisco for *Ca*. *hystrix*, *Cy*. *phanera* and *Cy*. *patelliformis*, and the distribution area of *P*_CW_ and *P*_R_ of these trees fell in the zone (Fig 4), suggesting that these tree leaves had insufficient nitrogen for Rubisco and cell walls [18]. Onoda et al. [7] and Takashima et al. [17] found a trade-off between nitrogen in cell walls and nitrogen in Rubisco in *Polygonum cuspidatum* and *Quercus* species, respectively. Zhang et al. also found this trade-off in *Mikania micrantha* and *Chromolaena odorata* [73]. They suggested that plants changed nitrogen allocation to increase either the rate or the duration of carbon assimilation. Hikosaka and Shigeno [15] considered this relationship unlikely to hold as a general rule; allocation of nitrogen to cell walls did not explain the variation in Rubisco. Harrison et al. [18] and Qing et al. [14] considered whether this relationship could exist when leaf nitrogen was deficient, and our results confirmed this. There is some other nitrogen in leaves apart from cell walls and Rubisco nitrogen, such as free amino acids [74], cyanogenic glycosides [75], lipids [17], inorganic nitrogen (NO_3_^–^, NH_4_^+^) [76], and so on. The other seven trees we studied may allocate a high percent of nitrogen to these functions (higher *P*_Other_, Table 4), and therefore, might weaken the correlation between Rubisco and cell wall nitrogen.

## Conclusion

We confirmed that PNUE of Fagaceae was significantly higher than that of Leguminosae, mainly attributed to a higher *P*_R_ and *P*_B_. LMA of Leguminosae species was lower than that of Fagaceae species, while there was no significant difference shown for *g*_m_, Fagaceae tree species may have greater chloroplast to total leaf surface area ratios and that offset the action of thicker cell walls on *g*_m_. Furthermore, weak negative relationship was between nitrogen allocation in cell walls and in Rubisco for *Ca*. *hystrix*, *Cy*. *phanera* and *Cy*. *patelliformis*, which might imply that nitrogen in the leaves was insufficient for both Rubisco and cell walls. In summary, our study concluded that higher PNUE might contribute to the dominance of most Fagaceae tree species in Jianfengling tropical montane rain forest.

## Supporting information

S1 Table Mesophyll conductance (*g*_m_) calculated by three methods in 10 Jianfengling tree species leaves *g*_m_ calculated by three methods (Harley, Ethier and Gu) were shown. Mean values (± SE) are shown (n = 5 for species and n = 25 for families). Different letters indicate significant differences between species and families (ANOVA, LSD test, *P* < 0.05). *F*-ratios with statistically significant values are denoted by * *P*<0.05, ** *P*<0.01, *** *P*<0.001.; data were measured in light-saturated and atmospheric CO_2_ concentrations of 380 μmol mol^–1^.
(DOCX)

S2 Table Chlorophyll concentration in 10 Jianfengling tree species leaves. Mean values (± SE) are shown (n = 5 for species and n = 25 for families). Different letters indicate significant differences between species and families (ANOVA, LSD test, *P* < 0.05). *F*-ratios with statistically significant values are denoted by * *P*<0.05, ** *P*<0.01, *** *P*<0.001. DW means the concentration of chlorophyll in dry mass.
(DOCX)
