# Supplementary material for: Fagaceae tree species allocate higher fraction of nitrogen to photosynthetic apparatus than Leguminosae in Jianfengling tropical montane rain forest, China
Source: PLoS One. 2018 Feb 1;13(2):e0192040. doi: 10.1371/journal.pone.0192040 (PMC5794133; doi:10.1371/journal.pone.0192040)
Supplement: S1 Table — Mean values (± SE) are shown (n = 5 for species and n = 25 for families). Different letters indicate significant differences between species and families (ANOVA, LSD test, P < 0.05). F-ratios with statistically significant values are denoted by * P<0.05, ** P<0.01, *** P<0.001.; data were measured in light-saturated and atmospheric CO2 concentrations of 380 μmol mol–1. (DOCX) [file pone.0192040.s001.docx]

**Table S1. Mesophyll conductance (*g*_m_) calculated by three methods in 10 Jianfengling tree species leaves**

| **Species** | **Families** | ***g*_m_(molCO_2_ m^–2^ s^–1^)** **Harley** | ***g*_m_(molCO_2_ m^–2^ s^–1^)Ethier** | ***g*_m_(molCO_2_ m^–2^ s^–1^) Gu** |
| --- | --- | --- | --- | --- |
| *O. fordiana* | Leguminosae | 0.023±0.004^def^ | 0.029±0.001^de^ | 0.028±0.001^def^ |
| *P. clypearia* |  | 0.034±0.005^cdef^ | 0.036± 0.005^cde^ | 0.038±0.005^cdef^ |
| *P. lucidum* |  | 0.047±0.007^bcde^ | 0.047± 0.005^bcde^ | 0.046±0.004^cd^ |
| *O. semicastrata* |  | 0.025±0.004^def^ | 0.028± 0.001^de^ | 0.029±0.001^def^ |
| *O. balansae* |  | 0.057±0.009^bcd^ | 0.060± 0.007^bc^ | 0.061±0.007^b^ |
| *L. fenzelianus* | Fagaceae | 0.026±0.004^def^ | 0.031± 0.004^cde^ | 0.030±0.003^def^ |
| *Ca. hystrix* |  | 0.029±0.005^cdef^ | 0.032± 0.016^cde^ | 0.032±0.002^def^ |
| *Ca. fissa* |  | 0.086±0.017^a^ | 0.105± 0.003^a^ | 0.082±0.004^a^ |
| *Cy. phanera* |  | 0.038±0.006^bcdef^ | 0.047± 0.005^bcd^ | 0.045±0.004^cd^ |
| *Cy. patelliformis* |  | 0.034±0.005^cdef^ | 0.038 ±0.004^cde^ | 0.035±0.005^cdef^ |
| *F* |  | 7.159^***^ | 13.433^***^ | 17.618^***^ |
|  | Leguminosae | 0.037±0.004^a^ | 0.040± 0.003^a^ | 0.040±0.003^a^ |
|  | Fagaceae | 0.043±0.006^a^ | 0.051± 0.007^a^ | 0.045±0.004^a^ |
|  | *F* | 0.652 | 2.183 | 0.777 |

*g*_m_ calculated by three methods (Harley, Ethier and Gu) were shown. Mean values (± SE) are shown (n=5 for species and n=25 for families). Different letters indicate significant differences between species and families (ANOVA, LSD test, *P* < 0.05). *F*-ratios with statistically significant values are denoted by ^*^ *P*<0.05, ^**^ *P*<0.01, ^***^ *P*<0.001.; data were measured in light-saturated and atmospheric CO_2_ concentrations of 380 μmol mol^–1^.
