# Supplementary material for: Fagaceae tree species allocate higher fraction of nitrogen to photosynthetic apparatus than Leguminosae in Jianfengling tropical montane rain forest, China
Source: PLoS One. 2018 Feb 1;13(2):e0192040. doi: 10.1371/journal.pone.0192040 (PMC5794133; doi:10.1371/journal.pone.0192040)
Supplement: S2 Table — Mean values (± SE) are shown (n = 5 for species and n = 25 for families). Different letters indicate significant differences between species and families (ANOVA, LSD test, P < 0.05). F-ratios with statistically significant values are denoted by * P<0.05, ** P<0.01, *** P<0.001. DW means the concentration of chlorophyll in dry mass. (DOCX) [file pone.0192040.s002.docx]

**Table S2.** **Chlorophyll concentration in 10 Jianfengling tree species leaves.**

| **Species** | **Families** | **Chl *a* (mg g^-1^ DW)** | **Chl *b* (mg g^-1^DW)** | **Chl *a*+*b* (mg g^-1^DW)** |
| --- | --- | --- | --- | --- |
| *O. fordiana* | Leguminosae | 0.47±0.09^bcde^ | 0.15±0.03^bcd^ | 0.62±0.10^bcd^ |
| *P. clypearia* |  | 0.46±0.02^bcde^ | 0.14±0.01^bcde^ | 0.60±0.03^bcde^ |
| *P. lucidum* |  | 0.78±0.09^a^ | 0.23±0.02^a^ | 1.01±0.10^a^ |
| *O. semicastrata* |  | 0.49±0.02^bcd^ | 0.14±0.01^bcd^ | 0.63±0.03^bcd^ |
| *O. balansae* |  | 0.50±0.05^bcd^ | 0.12±0.02^bcde^ | 0.62±0.06^bcd^ |
| *L. fenzelianus* | Fagaceae | 0.22±0.02^efg^ | 0.07±0.01^efg^ | 0.29±0.02^efg^ |
| *Ca. hystrix* |  | 0.24±0.01^defg^ | 0.08±0.01^defg^ | 0.31±0.01^defg^ |
| *Ca. fissa* |  | 0.44±0.07^bcde^ | 0.13±0.02^bcde^ | 0.57±0.09^bcde^ |
| *Cy. phanera* |  | 0.32±0.02c^defg^ | 0.10±0.01^cdefg^ | 0.42±0.03^cdefg^ |
| *Cy. patelliformis* |  | 0.37±0.05^bcdef^ | 0.12±0.01^bcdef^ | 0.49±0.06^bcdef^ |
| *F* |  | 9.276^***^ | 9.009^***^ | 9.930^***^ |
|  | Leguminosae | 0.54±0.04^a^ | 0.16±0.01^a^ | 0.70±0.04^a^ |
|  | Fagaceae | 0.32±0.02^b^ | 0.10±0.01^b^ | 0.42±0.03^b^ |
|  | *F* | 26.546^***^ | 22.585^***^ | 26.831^***^ |

Mean values (± SE) are shown (n=5 for species and n=25 for families). Different letters indicate significant differences between species and families (ANOVA, LSD test, *P* < 0.05). *F*-ratios with statistically significant values are denoted by ^*^ *P*<0.05, ^**^ *P*<0.01, ^***^ *P*<0.001. DW means the concentration of chlorophyll in dry mass.
